# Effect of Mindful Hypnotherapy on Psychological Distress: A Systematic Review and Meta-Analysis

**DOI:** 10.3390/bs16010107

**Published:** 2026-01-13

**Authors:** Victor Julián Padilla, Vanessa Muñiz, Katherine Scheffrahn, Gary Elkins

**Affiliations:** Department of Psychology and Neuroscience, Baylor University, Waco, TX 76701, USA; victor_padilla2@baylor.edu (V.J.P.); vanessa_muniz1@baylor.edu (V.M.); katherine_scheffrah2@baylor.edu (K.S.)

**Keywords:** psychological distress, mindfulness, hypnosis

## Abstract

Mindful hypnotherapy is an intervention that integrates hypnotic induction and direct suggestions (hypnosis) to increase mindfulness and reduce distress. In the present study, a systematic review and meta-analysis were conducted to investigate the effects of mindful hypnotherapy on psychological distress and mindfulness in adults. Further, the study evaluated the methodological quality of the included studies. A total of five publications of randomized controlled trials were identified. This meta-analysis found that, when compared to both waitlist and active control groups, mindful hypnotherapy had a large effect on the reduction in psychological distress (Hedges’ g = 0.61, 95% CI [0.10, 1.12], z = 2.36, *p* = 0.0188) and stress (Hedges’ g = 0.75, 95% CI [0.34, 1.16]; z = 3.58, *p* = 0.0003). Further, results also found mindful hypnotherapy had a large effect on increasing mindfulness (Hedges’ g = 1.38, SE = 0.28; 95% CI [0.83, 1.92]; z = 4.9146, *p* < 0.001). Recommendations include conducting research examining mindful hypnotherapy in a wider range of clinical problems (i.e., generalized anxiety disorder, depression, post-traumatic stress). In addition, clinical trials of mindful hypnotherapy should include active controls and measures of hypnotizability. Further research is also needed with diverse populations.

## 1. Introduction

Psychological distress has been described as a transient state of suffering characterized by symptoms of anxiety and depression that can occur in response to a stressor when an individual perceives their coping strategies and resources as insufficient (Horwitz, 2007; Ridner, 2004). Between 5% and 27% of the general population is estimated to experience high levels of psychological distress (Benzeval & Judge, 2001; Chittleborough et al., 2011; Gispert et al., 2003; Kuriyama et al., 2009; Phongsavan et al., 2006; Viertiö et al., 2021). Factors such as lack of social support, loneliness, chronic illness, and work dissatisfaction are associated with a higher prevalence of psychological distress. Adults with psychological distress have reported in qualitative interviews that their symptoms can interfere with their ability to carry out day-to-day duties and socialize with others (Arvidsdotter et al., 2016). Among the treatment options available, mindfulness-based interventions have been shown to be promising for improving symptoms of psychological distress (Karo et al., 2024; Park et al., 2020).

By practicing mindfulness, practitioners are encouraged to focus on their present experiences with an attitude of openness, curiosity, and nonjudgemental acceptance (Bishop et al., 2004). Research suggests that through regularly observing one’s passing thoughts and feelings nonjudgmentally during mindfulness practice, an individual can improve their ability to regulate their emotions by learning how to more intentionally respond to stressors (Baer, 2003; Hölzel et al., 2011). Popular mindfulness-based interventions such as mindfulness-based stress reduction and mindfulness-based cognitive therapy typically consist of eight two-and-a-half-hour group sessions and one full-day silent retreat conducted weekly with a trained facilitator (Kabat-Zinn et al., 1992; Miller et al., 1995; Teasdale et al., 2000). Participants in these programs are guided through group discussions, yoga, and sitting, walking, and body-scan meditation exercises.

While mindfulness-based interventions have been found to be moderately effective in reducing symptoms of anxiety and depression, concerns have been raised regarding mediation’s potentially distressing adverse side effects (Britton et al., 2021; Goldberg et al., 2018; Hofmann et al., 2010; Khoury et al., 2013). Facing one’s negative thoughts, feelings, and self-perceptions can be an uncomfortable and challenging experience for novice practitioners of meditation (Lomas et al., 2015). Furthermore, extensive self-practice may be necessary for individuals to experience significant improvements in their level of psychological distress (Bowles et al., 2022). These difficulties can be offset by the perceived benefits of mindfulness, but they can dissuade participants in mindfulness-based interventions from continuing with their self-practice and increase the likelihood of dropout (Taylor et al., 2012).

In recent years, it has been proposed that the integration of hypnotic inductions as well as therapeutic hypnotic suggestions and techniques may catalyze and facilitate the process of achieving mindfulness outcomes (Elkins et al., 2018; Lynn et al., 2006). Mindful hypnotherapy was originally developed by Elkins and Olendzki (2019) as a manualized eight-session intervention including weekly one-hour sessions and daily self-hypnosis guided home practice. Session topics included present moment awareness, nonjudgemental awareness of sensations, nonjudgemental awareness of thoughts and feelings, how to perform self-hypnosis, compassion for self and others, mindful awareness of values, integrating mindfulness into daily life, and transitioning to long-term practice.

Hypnosis is a state of consciousness marked by focused attention during which an individual may experience reduced peripheral awareness and a greater capacity for responding to suggestions for behavioral change (Elkins et al., 2015). Through the integration of hypnotic suggestions, it may be that individuals can be guided to attain a state of highly focused attention, respond to positive suggestions to deidentify with distressful thoughts, and experience positive outcomes such as mindfulness, compassion, and reduction in psychological distress (Lynn et al., 2008; Olendzki et al., 2020). Mindful hypnotherapy is an intervention that integrates hypnotic inductions, suggestion, and re-alerting techniques to assist in mindfulness practice for personal use or therapeutic purposes (Elkins & Olendzki, 2019).

While there have been several studies using mindful hypnotherapy, the effect of mindful hypnotherapy for reducing psychological distress and improving mindfulness has yet to be fully determined and there have been no prior systematic reviews or meta-analyses. The present study provides a systematic review and meta-analysis of mindful hypnotherapy research to achieve the following: (1) investigate the effects of mindful hypnotherapy interventions on psychological distress in adults, (2) investigate the effects of mindful hypnotherapy interventions on mindfulness in adults, and (3) evaluate the methodological quality of the included randomized controlled trials.

## 2. Materials and Methods

This systematic review and meta-analysis followed the Preferred Reporting Items for Systematic Reviews and Meta-Analyses (PRISMA) guidelines (Page et al., 2021). The protocol was pre-registered with the International Prospective Register of Systematic Reviews (PROSPERO) under registration number CRD42024617269.

### 2.1. Eligibility Criteria

The eligibility criteria for the current review were developed using the Population, Intervention, Comparison, Outcomes, and Study design (PICOS) framework (Cumpston et al., 2019). Studies were included if (P) the participants were adults aged 18 years or older; (I) the intervention was mindful hypnotherapy—defined as those that aimed to treat psychological or physiological conditions or concerns through the use of hypnotic suggestions and techniques to promote and facilitate mindfulness practice; (C) the comparison intervention was active, waitlist, or treatment as a usual control; (O) the outcomes were in domains related to psychological distress such as stress, anxiety, or depression, or related to mindfulness, such as acceptance or nonreactivity; and (S) the study design was a randomized controlled trial (RCT).

### 2.2. Study Selection and Data Extraction

A search was conducted on PubMed, PsycINFO, and Web of Science for articles published from the first available date up to when the search was conducted on 19 December 2024. The search strategies used for the three databases with the appropriate syntax and subject headings can be found in Supplement S1. Article screening and data extraction were carried out by two authors independently using the Covidence software for systematic review management; any discrepancies were discussed and any conflicts were resolved by a third author The following information was extracted from the included studies: (1) study characteristics (funding source, setting, authors’ names, year of publication), (2) methods (design, number of sessions, study length), (3) participants (sample size, inclusion and exclusion criteria, age, gender, race), (4) intervention (intervention session length, mode of delivery, number of participants allocated, adverse event reporting, adherence, home practice), and (5) outcomes (continuous measures of psychological distress, stress, anxiety, depression, and mindfulness).

### 2.3. Risk of Bias Assessment

Risk of bias was assessed for each of the included studies independently by the first and third author using Version Two of the Cochrane Risk of Bias tool (Sterne et al., 2019). The risk of bias was evaluated for each outcome from each of the included studies according to five domains: (1) bias from the randomization process, (2) bias from deviations from intended interventions, (3) bias from missing outcome data, (4) bias from measurement of the outcome, and (5) bias from selection of reported results. Each outcome was assessed as having a low risk of bias, some concerns, or a high risk of bias for each domain, and a total risk of bias assessment was performed for each of the included studies based on the assessments of their outcomes. Discrepancies in independent ratings were resolved in group discussions between all authors.

### 2.4. Quality of Evidence Assessment

The Grades of Recommendation, Assessment, Development, and Evaluation (GRADE) approach was taken to evaluate the quality of evidence from the included studies for psychological distress and mindfulness outcomes to inform clinical decision making (Guyatt et al., 2011). Outcomes from the included studies were rated based on their importance in clinical practice, and the findings for the selected outcomes were assessed based on study design, risk of bias, consistency, directness, and precision.

### 2.5. Statistical Analysis

The statistical analysis for this study’s meta-analysis was performed using R-studio version 2025.05.0+496. After screening, data were extracted and meta-analyses were performed for eight different outcome measures; these include psychological distress, stress, total mindfulness score, and subscale scores that capture five different facets of mindfulness (observing, describing, acting with awareness, nonjudging, and nonreactivity). The effect sizes reported for the included studies were converted into pre–post between-group Hedges’ g effect sizes. For studies that did not report an effect size, the baseline and post-intervention means, standard deviations, and number of participants in each arm were extracted to calculate the effect size using the *esc* package version 0.5.1 in R. In an included study that had three arms (mindful hypnotherapy with resistance training, resistance training alone, and waitlist control), the two groups that were used in our analyses were the mindful hypnotherapy arm and the active control (resistance training) arm. A random effects model was used to obtain a pooled effect size of Hedges’ g, 95% confidence intervals (95% CIs), and z and *p* statistics. A random effects model was used due to the small number of studies included, and the differences between included studies (e.g., the outcome measures used and differences in type of control group). In this study, an effect size below 0.5 was considered to be small, an effect size between 0.5 and 0.79 was considered to be medium, and an effect size above 0.8 was considered to be large (Cohen, 1988). Forest plots were produced for the three main outcome measures observed (psychological distress, stress, and total mindfulness scores). 

Cochrane’s Q test of heterogeneity and I^2^ index were used to assess heterogeneity. Based on recommended guidelines in the prior literature, a statistically significant Q value indicates heterogeneity of the effect sizes, whereas I^2^ values represent varying degrees of variance in effect sizes attributable to true between-study heterogeneity. An I^2^ value of 25% is considered low, 50% is considered medium, and 75% and higher is considered high heterogeneity (Higgins & Thompson, 2002).

## 3. Results

### 3.1. Study Inclusion

**Figure 1:**
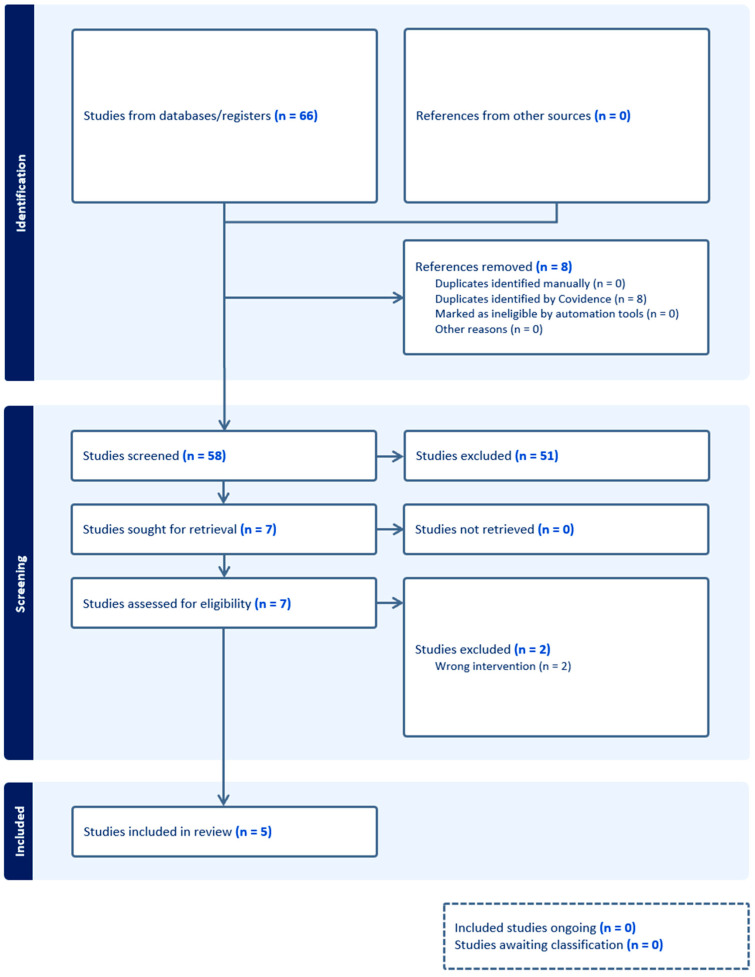
PRISMA flow chart.

A total of five publications were included in the systematic review and meta-analysis (Figure 1). All of the studies were randomized clinical trials with a parallel design. Two studies were excluded during full text screening. A pilot study by Hidderley and Holt (2004) on the use of autogenic training for stress in early cancer patients was excluded due to its lack of focus on suggestions for increasing mindfulness despite its use of a hypnotic-like state. The study by Stoerkel et al. (2018) on the use of a self-care toolkit for reducing anxiety in women with breast cancer was excluded given that the self-hypnosis audio track administered to participants did not include suggestions for mindfulness. The mindfulness meditation audio track in the toolkit was provided to participants separately. Of the studies included in the present review, three of the studies were conducted in the United States (Lin Latt et al., 2024; Olendzki et al., 2020; Slonena & Elkins, 2021), and two of the studies were conducted in Iran (Khazraee et al., 2023a, 2023b). Similarly, three of the studies were conducted in a university research lab setting (Lin Latt et al., 2024; Olendzki et al., 2020; Slonena & Elkins, 2021), and two of the studies were conducted in a hospital psychology clinic setting (Khazraee et al., 2023a, 2023b). Further study characteristics are reported in Table 1.

### 3.2. Participant Characteristics

The five studies included in this review included *N* = 209 participants. Of these, *n* = 105 were assigned to a mindful hypnotherapy intervention group, and *n* = 104 were assigned to a control group. The mean age reported from all studies combined was 23.92 years. The combined sample was predominantly female, with 190 (90.9%) being female. The racial and ethnic demographics of the combined sample were 34.4% White (*n* = 73), 32.5% Persian (*n* = 68), 12.0% Hispanic/Latino (*n* = 25), 8.1% Asian (*n* = 17), 4.3% Black (*n* = 9), 1.4% Mixed race/ethnicity (*n* = 3), and 0.5% Native American/Alaskan Native (*n* = 1). Table 2 shows demographic characteristics for each included study.

### 3.3. Intervention Characteristics

Olendzki et al. (2020) utilized a mindful hypnotherapy intervention design described in Elkins and Olendzki (2019), which was compared to a waitlist control. The intervention group attended sessions in a university research lab setting. Each session had a didactic teaching component as well as a hypnotic induction. The inductions were specifically designed for the topic of each weekly session. Each session, participants were given an audio with the topic for that week in order to practice mindful hypnotherapy at home. The topics for the eight weekly sessions were as follows: present-moment awareness, nonjudgmental awareness of the five senses, nonjudgmental awareness of thoughts and feelings, mindful hypnotherapy, compassion for self and others, awareness of personal values and meaning in life, integrated mindfulness, and termination/transition to long-term practice.

Slonena and Elkins (2021) compared a brief mindful hypnotherapy intervention to a cognitive training control group. Both the intervention and the control were delivered through 25 min audio recordings, with one being delivered after randomization and baseline, one being given to participants to use daily at home, and one delivered at the final, in-person session. The two groups’ recordings were matched in length, timing of instructions, silent rest periods, and attentional engagement, and had the same narrator. The brief mindful hypnotherapy intervention was adapted from the Olendzki et al. (2020) study. The mindful hypnotherapy intervention included a teaching component as well as a hypnotic induction. The intervention included suggestions for relaxation, resiliency to stress, present-moment awareness, acceptance, nonjudgmental awareness of thoughts and feelings, and compassion for self and others.

Khazraee et al.’s (2023a) mindful hypnotherapy intervention is based on that of Elkins and Olendzki (2019), with the protocol translated into Persian. In this study, the mindful hypnotherapy intervention group was compared to a waitlist control. The intervention was delivered in individual, in-person, weekly sessions by a clinical psychologist. The sessions included a didactic teaching component and hypnotic induction. The suggestions were individualized to each participant’s specific problems, needs, and goals. Participants were also given a prerecorded audio to use for mindful self-hypnosis at home. The sessions included suggestions for present-moment awareness, nonjudgmental awareness of bodily sensation, nonjudgmental awareness of thoughts and feelings, self-hypnosis, compassion for self and others, awareness of personal values and meaning in life, integrated mindfulness, and termination/transition to long-term practice.

In a second study, Khazraee et al. (2023b) investigated the effect of mindful hypnotherapy in a clinical population of individuals experiencing depression. In this study, the mindful hypnotherapy intervention was delivered in individual, in-person, weekly sessions by a clinical psychologist, and the intervention was compared to a waitlist control. The intervention followed the Elkins & Olendzki treatment manual for mindful hypnotherapy (Elkins & Olendzki, 2019).

Lin Latt et al. (2024) compared mindful hypnotherapy plus resistance training versus resistance training alone against a waitlist control group. Participants in the two groups involving resistance training were asked to do a training session three times a week for five weeks. Every third session was supervised by a researcher. The length of time for the sessions increased throughout the study, going from 45 min to 60 min by the conclusion. After a warmup, the training sessions included squat, press, and deadlift variations. Participants were asked to perform 2–4 sets of 8–12 repetitions with two-minute breaks between sets. The mindful hypnotherapy intervention group performed the same training routines as the resistance training only group, with the exception of five-minute mindful self-hypnosis sessions before and after the training. During the supervised sessions, the researcher delivered the mindful hypnotherapy in person; during the unsupervised sessions, participants used an audio recording with the intervention. The mindful hypnotherapy session used suggestions that were designed using Elkins and Olendzki (2019). Suggestions included focus of attention; nonjudgmental awareness of the present moment; guided imagery for the pre-training sessions; relaxation for the post-training sessions; curiosity with present-moment awareness; nonjudgmental awareness of sensations, thought, and feelings; integration of mindful self-hypnotherapy; increasing compassion; and having awareness of meaning in life.

Further information on the interventions and control groups for each study can be found in Table 3.

### 3.4. Data Synthesis from Meta-Analysis

#### 3.4.1. Psychological Distress

**Figure 2:**
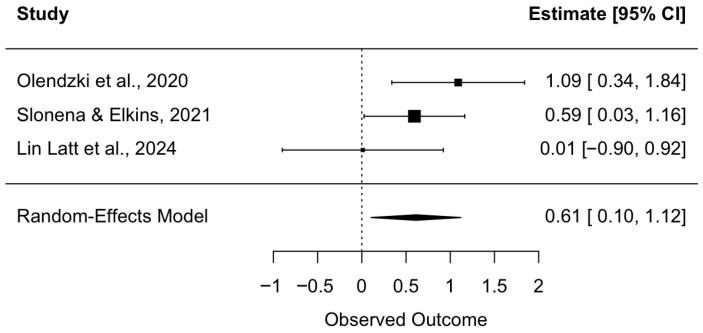
Forest plot of intervention’s effect on psychological distress. Each study’s individual effect size is represented by a square box. The horizontal line running through each box shows its 95% confidence interval. The size of each box is proportional to its weight on the overall effect estimate. The overall effect estimate and its 95% confidence interval are represented by a diamond at the bottom of the figure. The dashed vertical line is the line of no effect. If a confidence interval crosses this line, the effect is shown to be nonsignificant. *Note*: Olendzki et al. (2020) had a passive control group (waitlist control). Slonena and Elkins (2021) (cognitive training) and Lin Latt et al. (2024) (resistance training) had active control groups as comparators.

Of the included studies, three assessed psychological distress using a direct measure. This included the Psychological Distress Profile (PDP; Lin Latt et al., 2024; Olendzki et al., 2020) and the Subjective Units of Distress Scale (SUDS; Slonena & Elkins, 2021). A random effects meta-analysis was conducted on these studies to estimate the overall effects of a mindful hypnotherapy intervention on psychological distress. Olendzki et al. (2020) reported a statistically significant negative effect, g = −1.09; however, since all studies agreed that the intervention reduced distress, this effect size was recoded to ensure that all effect sizes indicated reduced distress in the intervention group, consistent with their findings. Slonena and Elkins (2021) found a statistically significant positive effect, with Hedges’ g = 0.59 and 95% CI [0.03, 1.16], while Lin Latt et al. (2024) reported a small positive but non-significant effect, with Hedges’ g = 0.01 and 95% CI [−0.90, 0.92]. The overall pooled effect size was medium and statistically significant, with Hedges’ g = 0.61, z = 2.36, *p* = 0.0188, and 95% CI [0.10, 1.12], suggesting consistent evidence of an effect across studies (see Figure 2). A test for heterogeneity showed Q(2) = 3.23, *p* = 0.1986, and I^2^ = 32.28%. Given that this analysis included three studies, the power to detect heterogeneity was limited (von Hippel, 2015).

#### 3.4.2. Stress

**Figure 3:**
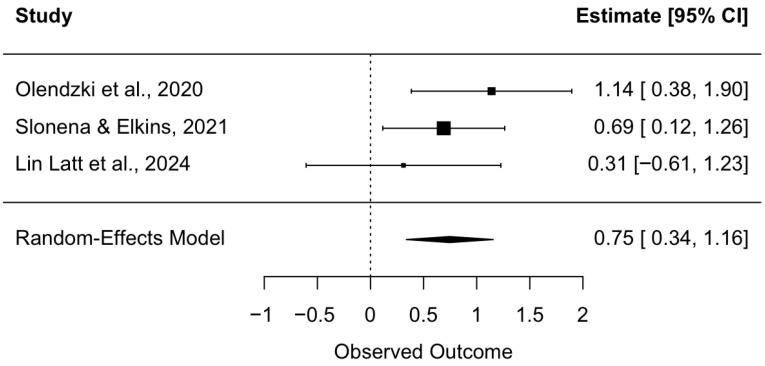
Forest plot of intervention’s effect on stress. Each study’s individual effect size is represented by a square box. The horizontal line running through each box shows its 95% confidence interval. The size of each box is proportional to its weight on the overall effect estimate. The overall effect estimate and its 95% confidence interval are represented by a diamond at the bottom of the figure. The dashed vertical line is the line of no effect. If a confidence interval crosses this line, the effect is shown to be nonsignificant. *Note*: Olendzki et al. (2020) had a passive control group (waitlist control). Slonena and Elkins (2021) (cognitive training) and Lin Latt et al. (2024) (resistance training) had active control groups as comparators.

A random effects meta-analysis was conducted to estimate the overall effects of a mindful hypnotherapy intervention on stress. A total of three studies were included in this analysis. These studies measured stress using either the Perceived Stress Scale (PSS; Lin Latt et al., 2024, Olendzki et al., 2020) or the Weekly Stress Inventory—Short Form (WSI-SF; Slonena & Elkins, 2021). Recalculation was performed on Olendzki et al. (2020), which reported a statistically significant negative effect, with Hedges’ g = −1.14, in favor of the intervention’s effect on reduced distress. This effect size was recoded to ensure that positive effect sizes indicated reduced distress in the intervention group, consistent with their findings. The pooled effect size was medium in magnitude and significant, indicating a reduction in stress post-intervention, with a Hedges’ g of 0.75 (z = 3.58, *p* = 0.0003) and a 95% confidence interval ranging from 0.34 to 1.16 (see Figure 3). Tests of heterogeneity revealed no evidence of between-study variability among studies, with Q(2) = 1.95, *p* = 0.38, and I^2^ = 0.0%. Given that this analysis included three studies, the power to detect heterogeneity was limited (von Hippel, 2015).

#### 3.4.3. Mindfulness

**Figure 4:**
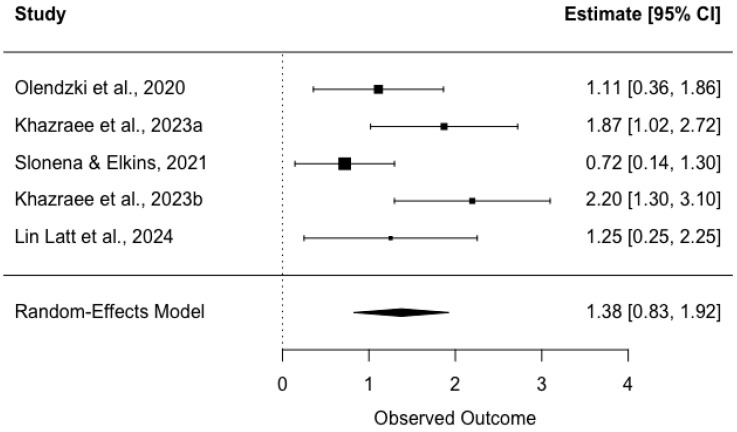
Forest plot of intervention’s effect on mindfulness. Each study’s individual effect size is represented by a square box. The horizontal line running through each box shows its 95% confidence interval. The size of each box is proportional to its weight on the overall effect estimate. The overall effect estimate and its 95% confidence interval are represented by a diamond at the bottom of the figure. The dashed vertical line is the line of no effect. If a confidence interval crosses this line, the effect is shown to be nonsignificant. *Note*: Studies with a passive control group (waitlist control) included Olendzki et al. (2020) and Khazraee et al. (2023a, 2023b). Studies with an active control group were Slonena and Elkins (2021) (cognitive training) and Lin Latt et al. (2024) (resistance training).

A random effects meta-analysis was conducted to estimate the overall effect of a hypnotherapy-delivered intervention on mindfulness across five independent studies (*k* = 5). As shown in Figure 4, the overall effect size was statistically significant (*z* = 4.9146, *p* < 0.001) and large with a Hedges’ g of 1.38 (*SE* = 0.28; 95% CI = 0.83 to 1.92). Examination of heterogeneity showed moderate between-study variability (I^2^ = 57.74%). A significant Q test for heterogeneity further confirmed the presence of variability among studies, with *Q*(4) = 9.65 and *p* = 0.047.

In addition, random effects models were carried out to examine each of the five facets of mindfulness as measured by the Five Facets Mindfulness Questionnaire (FFMQ) or the Kentucky Inventory of Mindfulness Skills (KIMS). From the aforementioned studies, three used the FFMQ (Olendzki et al., 2020; Khazraee et al., 2023b; Lin Latt et al., 2024) and Slonena and Elkins (2021) used the KIMS to assess mindfulness. The Khazraee et al. (2023a) study used the Acceptance and Commitment Questionnaire-II (AAQ-II) measure, along with Olendzki et al. (2020). Olendzki et al. (2020) only reported total pre–post results for the AAQ-II assessment and not for the FFMQ; however, findings from the FFMQ subscales were reported and included in Table 4.

### 3.5. Risk of Bias and Quality Assessment

**Figure 5:**
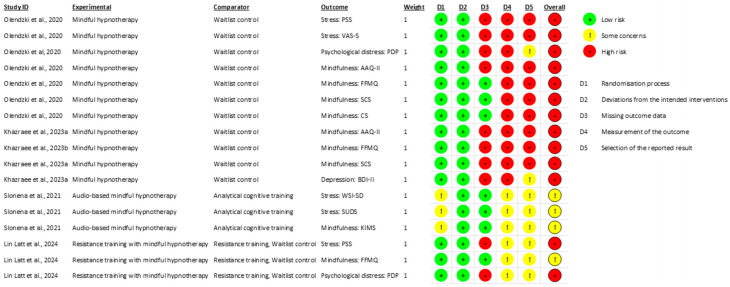
Risk of bias assessments per outcome within each study. Studies in the current review included Olendzki et al. (2020), Khazraee et al. (2023a), Khazraee et al. (2023b), Slonena and Elkins (2021); Lin Latt et al. (2024).

The findings from the risk of bias assessment are summarized in Figure 5. Four out of the five studies included in the present review (80%) had an outcome with a high risk of bias in at least one domain of the RoB2. There were some concerns in one study (20%) of there being bias from the randomization process. The risk of bias from deviations from intended interventions was low across all included studies. Four studies (80%) were identified as having a high risk of bias from missing outcome data. Three studies (60%) had a high risk of bias from outcome measurement. There was risk of bias in selecting the reported outcomes in published manuscripts in four studies (80%). The findings from the quality assessment following the GRADE approach for psychological distress and mindfulness outcomes are summarized in Table 5.

## 4. Discussion

The aim of the present systematic review and meta-analysis was to evaluate the effects of mindful hypnotherapy interventions on psychological distress and mindfulness in adults. This was the first meta-analysis to assess the methods and findings from randomized controlled trials on the efficacy of mindful hypnotherapy interventions. The results of the analysis showed that mindful hypnotherapy had a medium effect on self-reported psychological distress and stress. The effect of mindful hypnotherapy on depression, another outcome related to psychological distress, was investigated in only one study to date, demonstrating a large effect (Khazraee et al., 2023a). Additionally, it was found that mindful hypnotherapy had a large effect on improving mindfulness. Mindful hypnotherapy was shown to increase pre–post-intervention total FFMQ, KIMS, and AAQ-II scores compared to passive, resistance training, and analytical cognitive training comparison groups. An analysis of the effects of mindful hypnotherapy on the facets of mindfulness on the FFMQ and KIMS showed a large effect across all subscales.

The literature on mindful hypnotherapy has several limitations. Of the five studies identified in this review, two studies compared a mindful hypnotherapy intervention to an active control condition (Lin Latt et al., 2024; Slonena & Elkins, 2021). The remaining studies used waitlist control or treatment as the usual control. This is important to control for contact and the role of expectancies (Jensen et al., 2020). Further, to date, there have been no studies comparing mindful hypnotherapy to a mindfulness-only control.

It was noted that there was moderate to high heterogeneity in mindfulness outcomes. The findings of this review indicate that mindful hypnotherapy may be a promising intervention for managing symptoms of psychological distress and improving mindfulness in adults. The preliminary evidence presented in this review is in line with claims by the authors, who initially developed mindful hypnotherapy, that hypnosis can assist participants with achieving mindfulness outcomes (Elkins & Olendzki, 2019).

The findings of this review are limited, however, by the small number of included studies as well as the varied intervention protocols, comparison conditions, and follow-up periods used. Furthermore, the present meta-analysis was limited in its ability to evaluate potential publication bias given its small sample size. The literature on mindful hypnotherapy will need to be continually monitored to assess treatment effectiveness across different study contexts.

Recommendations include that research should examine mindful hypnotherapy in a wider range of clinical problems (i.e., generalized anxiety disorder, depression, and post-traumatic stress). In addition, clinical trials of mindful hypnotherapy should include active controls and measures of hypnotizability. It is also recommended that further research be conducted with diverse populations using fully powered randomized clinical trials. It is imperative that as the literature on mindful hypnotherapy interventions grows, researchers release pre-specified data analysis plans to ensure transparency in outcome measurement and analysis and reduce the risk of publication bias.

## 5. Conclusions

The results of the present review show that mindful hypnotherapy interventions are generally brief and largely effective in improving mindfulness in adults. Mindful hypnotherapy has a moderate effect in reducing symptoms of psychological distress. These findings support the integration of hypnotic suggestions into mindfulness practice as a promising avenue for future research on treatments for psychological distress. Further research is needed to investigate the efficacy of mindful hypnotherapy interventions for stress-related disorders (e.g., anxiety, depression, sleep, and pain) in diverse populations and settings in comparison to usual care and active control interventions.

## Figures and Tables

**Table 1:** Characteristics of included studies.

Study ID	Groups	Sponsorship Source	Total *N*	Retention	Reported Outcomes in Study
Olendzki et al. (2020)	Mindful hypnotherapy intervention, waitlist control	None reported	42	71.4% (30/42 completed)	Stress, psychological distress, mindfulness
Slonena and Elkins (2021)	Brief mindful hypnotherapy, cognitive training control	None reported	55	92.7% (51/55 completed)	Stress, mindfulness, psychological distress
Khazraee et al. (2023a)	Mindful hypnotherapy intervention, waitlist control	Taleghani Hospital Research Development Committee, Shahid Beheshti University of Medical Sciences, Tehran, Iran (Grant Number: 28489)	34	91.2.2% (31/34 completed)	Depression, mindfulness
Khazraee et al. (2023b)	Mindful hypnotherapy, waitlist control	Taleghani Hospital Research Development Committee, Shahid Beheshti University of Medical Sciences, Tehran, Iran (Grant Number: 28489)	34	91.2% (31/34 completed)	Mindfulness
Lin Latt et al. (2024)	Mindful self-hypnotherapy + resistance training, resistance training only, waitlist control	None reported	44	68.2% (30/44 completed)	Stress, psychological distress, mindfulness

**Table 2:** Participant characteristics.

Study ID	Mean Age (Years)	Gender	Race	Participants Allocated to Intervention
Olendzki et al. (2020)	19.6	35 (83.3%) female	White = 28 (66.7%); Hispanic/Latino = 8 (19.0%); Mixed race/ethnicity = 3 (7.1%); Black = 2 (4.8%); Asian = 2 (4.8%)	22
Slonena and Elkins (2021)	19.5	43 (78.2%) female	White = 34 (61.8%); Hispanic/Latino = 10 (18.2); Asian = 8 (14.5); Black = 2 (3.6%); Native American/Alaskan Native = 1 (1.8%)	33
Khazraee et al. (2023a)	32.06	34 (100%) female	Persian = 34 (100%)	17
Khazraee et al. (2023b)	32.06	34 (100%) female	Persian = 34 (100%)	17
Lin Latt et al. (2024)	21	44 (100%) female	White = 11 (30.4%); Black = 5 (16.7%); Hispanic/Latino = 7 (23.3%); Asian = 7 (23.3%)	16

**Table 3:** Intervention characteristics of included dtudies.

Study ID	Study Aims	Intervention Sessions	Comparator	Home Practice	Inclusion Criteria	Exclusion Criteria
Olendzki et al. (2020)	(1) Investigate feasibility of MH; (2) investigate impact of MH on stress, psychological distress, and mindfulness compared to WLC	8 weekly, 1 h, in-person sessions	Waitlist control	Instructed to practice daily using home practice CD	(1) Proficiency in English; (2) at least 18 years of age; (3) self-identify as being highly stressed, as indicated by a score of 50% or higher on a visual analog scale measuring overall stress	(1) Diagnostic indicators or a history of borderline personality disorder, psychosis, or schizophrenia
Slonena and Elkins (2021)	(1) Investigate impact of a brief MH intervention on stress reactivity relative to an active control condition; (2) compare brief MH intervention to active control in relaxation, mindfulness, intervention satisfaction, and home practice adherence	2 weekly in-person sessions	Cognitive training (active control)	Instructed to listen to audio recording daily for one week	(1) At least 18 years of age; (2) proficient in English; (3) no prior experience with mindfulness or clinical hypnosis; (4) score > 18 on PSS	(1) Individuals with diagnostic indicators or a history of borderline personality disorder or psychosis; (2) those currently in psychotherapy treatment for major depressive disorder
Khazraee et al. (2023a)	Investigate the effectiveness of MH for depression, self-compassion, and psychological inflexibility in females with MDD	8 weekly, 1 h, in-person sessions	Waitlist control	Given daily practice audio recordings based on the content presented that week	(1) Score > 28 on BDI-II, indicating severe levels of depression; (2) diagnosis of MDD, according to the DSM-5; (3) between 18 and 50 years old; (4) having at the least a diploma from a high school/secondary school; (5) if receiving psychiatric medication, the dosage and type of medication were stable in the last three months before the start of the study and remained steady during the study period; (6) available for 8 weekly sessions	(1) Unwillingness to continue treatment; (2) serious suicidal thoughts and any plan(s) to attempt suicide; (3) receiving psychological interventions in the last 6 months; (4) participation in another psychological intervention at the same time as study; (5) recent substance abuse; (6) diagnostic indicators or history of borderline personality disorder, bipolar disorder, psychosis, or schizophrenia
Khazraee et al. (2023b)	Investigate the effectiveness of MH intervention on difficulties in emotion regulation, mindfulness, and mental health in patients with MDD	8 weekly, 1 h, in-person sessions	Waitlist control	Given audio recording of session material	(1) Score > 28 on BDI-II, indicating severe levels of depression; (2) diagnosis of MDD, according to the DSM-5; (3) between 18 and 50 years old; (4) having at the least a diploma from a high school/secondary school; (5) if receiving psychiatric medication, the dosage and type of medication were stable in the last three months before the start of the study and remained steady during the study period; (6) available for 8 weekly sessions	(1) Unwillingness to continue treatment; (2) serious suicidal thoughts and any plan(s) to attempt suicide; (3) receiving psychological interventions in the last 6 months; (4) participation in another psychological intervention at the same time as study; (5) recent substance abuse; (6) diagnostic indicators or history of borderline personality disorder, bipolar disorder, psychosis, or schizophrenia
Lin Latt et al. (2024)	(1) Investigate impact of mindful self-hypnotherapy combined with resistance training on perceived stress as measured by the PSS; (2) investigate whether this combination can improve psychological well-being and strength gains	5 min hypnotherapy before and after the 15 RT sessions which occurred 3× per week for five weeks. A total of 5 sessions were performed with a supervisor, and 10 were performed on their own	Resistance training only (active control) and waitlist control	Given two self-guided 45–60 min sessions	(1) Full-time college students; (2) between 18 and 29 years old; (3) able to perform resistance training w/o health complications; (4) no previous experience of regular physical exercise in the past 6 months	(1) Previous diagnosis of serious psychiatric illnesses (e.g., bipolar disorder, borderline personality disorder, psychosis, or schizophrenia); (2) diagnosis of major depressive disorder in the past 5 years; (3) current substance use (including smoking cigarettes); (4) if participants were taking anti-anxiety or anti-depressant medication, they were asked to not change their dose for one month prior to and during the intervention; to not use additional hypnosis or meditation during the intervention; and to not begin taking supplements that could impact strength throughout the course of the intervention

**Table 4:** Mindfulness subscale analysis.

	Hedges’ g	95% CI	*p*	Q	P	I^2^
Observing	0.849	0.1866, 1.5110	0.012	10.07	0.02	67.76%
Describing	0.649	0.2228, 1.0762	0.0029	4.05	0.26	27.96%
Acting with Awareness	0.890	0.529, 1.252	<0.0001	1.72	0.63	0.0%
Nonjudging	0.95	0.1704, 1.7367	0.017	10.93	0.01	76.31%
Nonreactivity	1.18	0.096, 2.266	0.03	9.04	0.01	79.37%

**Table 5:** GRADE summary: mindful hypnotherapy interventions for psychological distress and mindfulness.

Patient or population: Adults aged 18 years of age or older Intervention: Mindful hypnotherapy interventions Comparator: Active, waitlist, or treatment as usual control Outcome: Symptoms of psychological distress; mindfulness Study design: Randomized control trials				
Outcomes	Effect size Hedges’ g	Significance	N participants	Quality of evidence (GRADE)
Psychological distress PDP, SUDS	Hedges’ g = 0.61, 95% CI [0.10, 1.12]	*p* = 0.018	141	⊕⊕⊖⊖ Low ^1,2,3,4^
Stress PSS, WSI-SF	Hedges’ g = 0.75, 95% CI [0.34, 1.16]	*p* = 0.0003	175	⊕⊕⊖⊖ Low ^1,2,3,4^
Mindfulness AAQ-II, FFMQ, KIMS	Hedges’ g = 1.38, 95% CI [0.83, 1.92]	*p* < 0.001	209	⊕⊕⊕⊖ Moderate ^1,3,4,5,6,7^

*Note*: PDP = Psychological Distress Profile; SUDS = Subjective Units of Distress Scale; PSS = Perceived Stress Scale; WSI-SF = Weekly Stress Inventory—Short Form; AAQ-II = Acceptance and Action Questionnaire II; FFMQ = Five Facets Mindfulness Questionnaire; KIMS = Kentucky Inventory of Mindfulness Skills. GRADE Working Group grades of evidence: ⊕⊕⊕⊕ High: We are very confident that the true effect lies close to that of the estimate of the effect. ⊕⊕⊕⊖ Moderate: We are moderately confident in the estimate of effect: the true effect is likely to be close to the estimate of effect, but has the possibility to be substantially different. ⊕⊕⊖⊖ Low: Our confidence in the effect is limited: the true effect may be substantially different from the estimate of the effect. ⊕⊖⊖⊖ Very low: We have very little confidence in the effect estimate: the true effect is likely to be substantially different from the estimate of effect. ^1^. High risk due to issues with allocation concealment and blinding self-report measures. ^2^. There was low heterogeneity among the studies included, but it was noted that this may have been in part due to the small number of studies in the present review. ^3^. Indirectness due to differences in intervention duration and modality. ^4^. Wide confidence interval can be accounted for by the sample size. ^5^. There was moderate heterogeneity among the studies included. ^6^. Indirectness due to measure of inflexibility being used to assess mindfulness. ^7^. Effect size was very large.

## Data Availability

The data and syntax used to reach the conclusions of the present review are available upon request from the authors.

## Supplementary Materials

The following supporting information can be downloaded at https://www.mdpi.com/article/10.3390/bs16010107/s1: Supplement S1: Search strategy by database.
