# Supplementary material for: Effect of Mindful Hypnotherapy on Psychological Distress: A Systematic Review and Meta-Analysis
Source: Behav Sci (Basel). 2026 Jan 13;16(1):107. doi: 10.3390/bs16010107 (PMC12837898; doi:10.3390/bs16010107)
Supplement: Supplementary file 1 [file behavsci-16-00107-s001.zip › Supplement 1_Search strategy.pdf]

**Supplement S1**

**Search strategy by database**

**PubMed**

1. “randomized controlled trial*”[Title/Abstract] OR “randomized controlled trial”[Publication Type] OR randomized controlled trials as topic[MeSH Terms] OR rct[Title/Abstract] OR random*[Title/Abstract] OR random allocation[MeSH Terms] OR “clinical trial”[Title/Abstract] OR (clinical[Title/Abstract] AND trial[Title/Abstract]) OR clinical trials as topic[MeSH Terms] OR clinical trial[Publication Type]
2. hypnosis[Title/Abstract] OR hypnosis[MeSH] OR hypnotherap*[Title/Abstract]
3. mindful*[Title/Abstract] OR mindfulness[MeSH] OR meditat*[Title/Abstract] OR meditation[MeSH] OR “mindfulness based-intervention”[Title/Abstract] OR mbi[Title/Abstract] OR “mindfulness-based stress reduction”[Title/Abstract] OR mbsr[Title/Abstract] OR “mindfulness-based cognitive therapy”[Title/Abstract] OR mbct[Title/Abstract]
4. “psychological distress”[Title/Abstract] OR psychological distress [MeSH] OR “emotional distress”[Title/Abstract] OR stress[Title/Abstract] OR anxiety[Title/Abstract] OR anxiety[MeSH] OR depression[Title/Abstract] OR depression[MeSH] OR depressive[Title/Abstract]
5. #1 AND #2 AND #3 AND #4

**APA PsycINFO**

1. TI (“randomized controlled trial” OR random* OR “clinical trial”)
2. AB (“randomized controlled trial” OR rct OR random* OR “clinical trial”)

3. TI (hypnosis OR hypnotherapy*)
4. AB (hypnosis OR hypnotherapy*)
5. TI (mindful* OR meditat* OR “mindfulness based-intervention” OR “mindfulness-based stress reduction” OR “mindfulness-based cognitive therapy”)
6. AB (mindful* OR meditat* OR “mindfulness based-intervention” OR mbi OR “mindfulness-based stress reduction” OR mbsr OR “mindfulness-based cognitive therapy” OR mbct)
7. TI (“psychological distress” OR “emotional distress” OR distress OR stress OR anxiety OR depression OR depressive)
8. AB (“psychological distress” OR “emotional distress” OR distress OR stress OR anxiety OR depression OR depressive)
9. S1 AND S2 AND S3 AND S4 AND S5 AND S6 AND S7 AND S8

**Web of Science**

1. TI=(“randomized controlled trial” OR random* OR “clinical trial”)
2. AB=(“randomized controlled trial” OR rct OR random* OR “clinical trial”)
3. TI=(hypnosis OR hypnotherapy*)
4. AB=(hypnosis OR hypnotherapy*)
5. TI=(mindful* OR meditat* OR “mindfulness based-intervention” OR “mindfulness-based stress reduction” OR “mindfulness-based cognitive therapy”)
6. AB=(mindful* OR meditat* OR “mindfulness based-intervention” OR mbi OR “mindfulness-based stress reduction” OR mbsr OR “mindfulness-based cognitive therapy” OR mbct)

7. TI=(“psychological distress” OR “emotional distress” OR distress OR stress OR anxiety OR depression OR depressive)
8. AB=(“psychological distress” OR “emotional distress” OR distress OR stress OR anxiety OR depression OR depressive)
9. #1 AND #2 AND #3 AND #4 AND #5 AND #6 AND #7 AND #8
